# Antibiotic stewardship practices at community living centers for veterans

**DOI:** 10.1017/ash.2026.10387

**Published:** 2026-05-11

**Authors:** Reinaldo Perez, Robin Jump, John D. Markley, Allison Kelly, Andrew Chou, Daniel J. Livorsi

**Affiliations:** 1 Duke University, Durham, USA; 2 Durham VA Medical Center, USA; 3 University of Pittsburgh School of Medicine, USA; 4 VA Pittsburgh Healthcare System, USA; 5 Richmond VAMC: Hunter Holmes McGuire VA Medical Center, USA; 6 Cincinnati VAMC: Cincinnati VA Medical Center, USA; 7 VA New England Healthcare System, USA; 8 Iowa City VAMC: Iowa City VA Medical Center, USA

## Abstract

We conducted a survey assessing antibiotic stewardship activities and resources in Veterans Health Administration Community Living Centers, which provide skilled nursing and residential care. We found high rates of implementation of cornerstone stewardship practices and opportunities for increased stewardship intervention with greater informatics support.

## Introduction

The US Centers for Medicare and Medicaid Services mandated implementation of antibiotic stewardship programs in long-term care facilities, including nursing homes, in 2017.^1^ Since that time, several studies have evaluated the adoption of the Centers for Disease Control and Prevention’s (CDC) core elements of antibiotic stewardship in nursing homes, or assessed the effectiveness of a particular stewardship strategy.^2–4^

Few studies specifically assessed Veterans Affairs (VA) nursing homes, termed Community Living Centers (CLCs). Like community nursing homes, CLCs offer both skilled nursing and residential care. CLCs differ, however, in being part of larger VA medical centers (VAMCs), which have robust infrastructure including on-site pharmacy, radiology, and clinical laboratory services. In 2023, the VA issued an unfunded directive recommending at least 0.25 full-time equivalents (FTEs) of a medical provider and pharmacist specifically in support of antibiotic stewardship in CLCs.^5^ We surveyed VAMCs to assess contemporary antibiotic stewardship practices in CLCs.

## Methods

We created a novel 22-question survey to evaluate antibiotic stewardship activities and the personnel involved specific to VA CLCs (Supplement). The survey was pilot tested at 5 VAMCs via REDCap in February 2024. The finalized survey was then distributed to all VAMCs using an internal antibiotic stewardship listserv on 5/20/24 and 6/28/24. On 7/11/24, targeted e-mails were sent to regional stewardship leads (n = 18) to encourage participation. Participants in the VA’s national antibiotic stewardship webinars were reminded about the survey during the May and August sessions. The survey was closed on 9/6/2024. The survey and data collection processes were deemed non-human participants’ research by the University of Iowa’s Institutional Review Board.

## Results

Ninety-nine of the 145 VAMCs completed surveys (response rate = 71%), of which 85 reported having a CLC and are the focus of this report. Stewardship physicians and pharmacists completed 50 (59%) surveys while other pharmacists completed 28 (32%), CLC medical directors 5 (6%) and other personnel completed 2 (2%). Surveyed CLCs had a median of 67 beds (Interquartile Range (IQR) 40–121) with a median of 2 physicians (IQR 1–4) and 3 advanced practice providers (IQR 2–4) acting as primary ordering clinicians. 63 (74%) facilities were within walking distance of their associated VAMC.

Resources available for antibiotic stewardship activities in the CLC varied across responding sites. Seventeen (20%) facilities had dedicated FTEs for an infectious diseases (ID)-trained pharmacist, 32 (38%) for a pharmacist without ID training, 13 (15%) for an ID physician, and 20 (24%) for a provider without ID training. Thirty-four sites (40%) reported no FTEs for stewardship personnel. Seventy-seven (91%) facilities had a microbiology lab at their VAMC, and all sites reported access to ID consultations in-person or through telehealth.

Most CLCs reported using common stewardship strategies, including using preauthorization for targeted antibiotics (66; 78%), facility-specific antibiotic treatment guidelines (58; 68%), prospective audit and feedback (51; 60%), and diagnostic stewardship (47; 55%).

Metrics tracked by CLC stewardship programs also varied. Seventy-seven (91%) respondents reported monitoring *Clostridioides difficile* infections, 63 (74%) tracked multidrug-resistant organism infections, 51 (60%) measured antibiotic days of therapy, 12 (14%) tracked rates of new antibiotic starts, 12 (14%) quantified adherence to local treatment guidelines, and 6 (7%) performed point prevalence surveys of antibiotic use. In terms of reporting of these data, 42 (49%) facilities described generating facility-specific reports on CLC-related antibiotic use and outcomes. These data were generally reported at stewardship committee meetings or other leadership meetings. Only 17 (20%) facilities shared these data with CLC providers and staff.

Respondents identified several barriers to improvement in antibiotic use at CLCs. Themes included lack of funding and resources (54%), lack of personnel (16%), and cultural resistance to change (16%) (Table 1).

**Table 1.** Barriers to improvement in antibiotic use at 85 VA CLCs

Barrier	Percent of respondents agreeing n (%)	Representative quotations
Lack of funding and resources	46 (54)	*“Need dedicated IT personnel for data reporting”*
Lack of personnel	14 (16)	*“Part time ID physician, ASP pharmacist also covers internal medicine, CLC pharmacist w/o FTE for ASP”*
Providers resistant to stewardship strategy	14 (16)	*“Resistance regarding urinary tract infections and using the proper documentation prior to obtaining a urine culture”*
Lack of priority from hospital leadership	13 (15)	–
Lack of ID support	10 (12)	*“Difficult to staff [an on-site ID specialist] in rural areas”*
No barriers	19 (22)	–
Other	16 (19)	*“CAC/ADPAC^a^ support to build and maintain order menus”* *“Limited data mining capabilities for CLC specific data”* *“Need better data pulls/report builds/dashboards specifically for the CLC if not comparing to other sites, then being a self comparator over time.”*

a CAC, clinical application coordinator, ADPAC, automated data processing application coordinator.

## Discussion

Our national survey of VA CLCs provides a valuable assessment of the current state of stewardship in this unique care setting. We observed high uptake of cornerstone antibiotic stewardship strategies, including preauthorization, prospective audit and feedback, and local treatment guidelines. However, opportunities remain to develop and standardize quality measures used for internal tracking and benchmarking. In addition, there are opportunities to improve the frequency of giving feedback to prescribers. The survey also identified perceived barriers preventing programs from strengthening antibiotic stewardship practices. Notably, 4 out of every 10 CLCs reported the lack of dedicated FTEs to support stewardship personnel, which indicates adequate funding for stewardship was absent at a large proportion of responding sites.

Prior studies into stewardship in the postacute care setting have largely focused on community nursing homes. These studies showed comparatively lower levels of involvement by trained antibiotic stewards and less utilization of key strategies such as preauthorization or prospective audit and feedback.^2,3^ In contrast, VA CLCs may benefit from a national directive mandating pharmacy and physician support for stewardship efforts. The synergistic relationship between VAMCs and their CLCs could serve as a model for partnership between large health systems and community nursing homes. Prior work within the VA has demonstrated the efficacy of telehealth-supported stewardship activities in the postacute care setting; this could serve as a model for private sector nursing homes.^6^

Our survey additionally sheds light on some limitations affecting antibiotic stewardship in CLCs. The most consistent theme reported by respondents was a lack of support for information technology and data analytic needs. A lack of informatics support could limit the implementation of interventions that leverage the electronic medical record or data monitoring specific to a new stewardship strategy. The VA national office has created an interactive dashboard to access antibiotic use data in CLCs, and further research will be needed to clarify if stewards are unaware of these resources or find them to be inadequate. In the private sector, acute care hospitals are being encouraged to submit antibiotic use data to the National Healthcare Safety Network allowing for generation of reports on antibiotic use and interfacility benchmarking. Encouraging data submission by long-term care facilities or otherwise centralizing data analytics through university and private partnerships may be a path to further improvement. At the current time, however, there are no validated quality measures on antibiotic use for stewardship programs in long-term care facilities to track.

Our study has limitations. VA CLCs operate within the largest integrated healthcare system in the US and differ from other nursing homes. Additionally, our survey was voluntary and completed by staff from variable disciplines, potentially introducing significant variability into the responses. Finally, our survey was not a previously validated tool and may be an imperfect measure of day-to-day stewardship activities in VA CLCs. Despite these limitations, we feel our survey adds value in describing the current landscape and guiding potential future projects for VA.

The CDC estimates that over 1.3 million people in the United States live in nursing homes with this population expected to grow rapidly as the population ages.^7^ Over time, improving antibiotic stewardship practices in long-term care settings will only increase in importance. Our survey emphasizes the benefits of nursing home partnership with acute care facilities. It further highlights the need for centralized informatics support to facilitate standardized metrics, benchmark evaluation, and change. Antibiotic stewardship has always been a team sport, and this collaborative spirit is also essential in the long-term care setting.

## Supporting information

10.1017/ash.2026.10387.sm001 Perez et al. supplementary material Perez et al. supplementary material

## Supplementary material

The supplementary material for this article can be found at https://doi.org/10.1017/ash.2026.10387.
