# Supplementary material for: Antibiotic stewardship practices at community living centers for veterans
Source: Antimicrob Steward Healthc Epidemiol. 2026 May 11;6(1):e127. doi: 10.1017/ash.2026.10387 (PMC13162059; doi:10.1017/ash.2026.10387)
Supplement: Perez et al. supplementary material [file S2732494X26103878sup001.pdf]

Survey of antibiotic stewardship practices in VA Community Living Centers

This survey seeks to gather information about antibiotic stewardship resources and processes in Community Living Centers (CLCs) across the Veterans Health Administration (VA). Your responses will help the VA's National Antimicrobial Stewardship Taskforce plan future outreach efforts specific to CLCs.

We ask that a person knowledgeable about antibiotic stewardship at your CLC respond to the survey.

For this survey, a CLC will be defined as a designated unit within a VA medical center that provides post-acute and long-term care, including dementia and hospice care. Spinal cord injury units will not be considered as being part of a CLC.

1a Name of survey respondent

____

1b Role of survey respondent

____

2a What is the location of your medical center? Please enter the city and state.

____

2b What VISN is your medical center part of?

- ◯ 1
- ◯ 2
- ◯ 4
- ◯ 5
- ◯ 6
- ◯ 7
- ◯ 8
- ◯ 9
- ◯ 10
- ◯ 12
- ◯ 15
- ◯ 16
- ◯ 17
- ◯ 19
- ◯ 20
- ◯ 21
- ◯ 22
- ◯ 23

3a Is there a CLC associated with your medical center?

- ◯ Yes
- ◯ No

3b Is the primary VA medical center within walking distance of the CLC units?

- ◯ Yes
- ◯ No

4 During FY2024, how many licensed CLC beds does your facility have? Estimate to an integer.

____

5 During FY2024, what has been your estimated daily census of CLC residents? Estimate to the nearest integer. __________________________________

Please estimate the percentage of residents at your CLC during FY2024 who have received the following types of care.

		None	1-25%	25-49%	50-74%	75-100%
6a	Post-acute (skilled) care after a hospital stay	◯	◯	◯	◯	◯
6b	Long-term (residential) care	◯	◯	◯	◯	◯
6c	Short-stay continuous care for placement	◯	◯	◯	◯	◯
6d	Long-stay continuous care	◯	◯	◯	◯	◯
6e	Respite care	◯	◯	◯	◯	◯
6f	Dementia care	◯	◯	◯	◯	◯
6g	Hospice care	◯	◯	◯	◯	◯
6h	Other care	◯	◯	◯	◯	◯

6i Please describe the Other type of care you referred to above. __________________________________

7a How often does a licensed independent practitioner (i.e. physician, NP, PA) perform a routine bedside assessment on short-stay residents at your CLC and document their management plan in the EMR?

- ◯ Every weekday (M-F)
- ◯ At least twice a week but less often than every weekday
- ◯ Once a week
- ◯ 2-3 times/month
- ◯ Once a month
- ◯ Other. Please explain below.

7b How often does a licensed independent practitioner (i.e. physician, NP, PA) perform a routine bedside assessment on long-stay residents at your CLC and document their management plan in the EMR?

- ◯ Every weekday (M-F)
- ◯ At least twice a week but less often than every weekday
- ◯ Once a week
- ◯ 2-3 times/month
- ◯ Once a month
- ◯ Other. Please explain below.

7c Please explain your Other response about the frequency of bedside assessments. __________________________________

8 Please indicate how many physicians (MD/DO) are currently responsible for antibiotic-prescribing at your CLC during working hours (e.g., 8a-5p on weekdays). __________________________________

9 Please indicate how many advance practitioners (NP/PA) are currently responsible for antibiotic prescribing at your CLC during working hours (e.g., 8a-5p on weekdays). __________________________________

10a During off-hours, who manages acute issues that arise in CLC residents? (Check all that apply)

- ☐ On-site VA staff provider who answers some issues in-person and some by phone
- ☐ On-site contract provider (i.e., not VA staff) who moonlights
- ☐ VA telehealth provider who is always off-site
- ☐ Contract telehealth who is always off-site provider (i.e., not VA staff) who moonlights
- ☐ VA Emergency Department
- ☐ Non-VA Emergency Department
- ☐ Other. Please explain below.

10b Other process for managing acute issues during off-hours (please describe)

____

Microbiology and Infectious Disease Expertise in your CLC

11 Does the CLC have access to a microbiology laboratory at the associated VA medical center?

◯ Yes ◯ No

12 Does your CLC have access to a VA-staffed inpatient Infectious Disease (ID) Consultation Service? Check all that apply.

- ☐ Yes, in-person VA-staffed ID consults are available to see CLC residents in the CLC
- ☐ Yes, in-person VA-staffed ID consults are available to see CLC residents in the outpatient ID clinic
- ☐ Yes, VA-staffed ID consults are available but only via telehealth (e.g., chart reviews or videoconferencing with CLC residents)
- ☐ No, VA-staffed ID consults are not available

13a When a patient is discharged from a hospital and sent to your CLC for a prolonged course of intravenous antibiotics, who usually monitors the Veteran's treatment course? Check all that apply.

- ☐ CLC providers
- ☐ The ID service at our facility (i.e., VA staff)
- ☐ Our facility's outpatient parenteral antimicrobial therapy (OPAT) service
- ☐ The non-VA ID provider who recommended the parenteral antibiotics
- ☐ Pharmacist
- ☐ Other. Please explain below.

13b Please explain your Other response about monitoring prolonged courses of intravenous antibiotics.

____

Please specify which of the following roles are responsible for supporting antibiotic stewardship in your CLC.
--An ID-trained pharmacist is defined as someone who completed a pharmacy post-graduate year 2 residency or fellowship in Infectious Diseases.
--An ID-trained physician is defined as someone who has completed an ID fellowship training program.
--Day-to-day stewardship activities may include prospective audit-and-feedback and reviewing prior authorization requests.
--Stewardship program oversight may include reviewing antibiotic use trends, preparing annual reports, and developing antibiotic-prescribing guidelines.

		Day-to-day stewardship activities	Stewardship program oversight	Not applicable
14a	ID-trained pharmacist	☐	☐	☐
14b	Non-ID pharmacist	☐	☐	☐
14c	ID-trained physician	☐	☐	☐
14d	Medical provider without ID training	☐	☐	☐
14e	Other (please explain below)	☐	☐	☐

14f Please explain who the Other staff member is who is involved in antibiotic stewardship efforts. __________________________________

For each of the following roles, are there FTEEs (full time equivalent employee) specifically allocated for antibiotic stewardship in the CLC?

--An ID-trained pharmacist is defined as someone who completed a pharmacy post-graduate year 2 residency or fellowship in Infectious Diseases.
--An ID-trained physician is defined as someone who has completed an ID fellowship training program.

		Yes, there are FTEEs for stewardship in CLC	No, there are no FTEEs for stewardship in CLC
15a	ID-trained pharmacist	○	○
15b	Non-ID pharmacist	○	○
15c	ID-trained physician	○	○
15d	Medical provider without ID training	○	○
15e	Other (please explain below)	○	○

15f Please explain who the Other staff member is who is involved in antibiotic stewardship efforts. __________________________________

Leadership support for antibiotic stewardship in your CLC

16a Identify the different ways that your medical center's leadership demonstrates support for antibiotic stewardship in your CLC. Check all that apply.

- ☐ Written statement of leadership support to improve antibiotic use
- ☐ Antibiotic stewardship duties included in the Medical Director's position description
- ☐ Antibiotic stewardship duties included in the Director of Nursing's position description
- ☐ Leadership monitors whether antibiotic stewardship policies are followed
- ☐ Antibiotic use and resistance data are reviewed in quality assurance meetings
- ☐ Other. Please explain below.
- ☐ None of the above.

16b Please describe your "Other" response for types of leadership support.

____

Antibiotic stewardship and diagnostic stewardship practices in your CLC

17a Which of the following policies or processes has your CLC implemented to improve antibiotic use? Check all that apply.

- ☐ Require prescribers to document a dose, duration, and an indication for all antibiotic prescriptions
- ☐ Facility-specific algorithms for appropriate diagnostic testing (e.g., obtaining cultures) for specific infections
- ☐ Facility-specific antibiotic treatment recommendations for common infections
- ☐ Prospective review of antibiotic agents listed on the medication formulary
- ☐ Preauthorization process for targeted antibiotics
- ☐ Other. Please explain below.
- ☐ None of the above.

17b If there is a routine process for prospective review of antibiotics on your CLC's formulary, which antibiotic agents are monitored in this manner? Please explain.

____

17c If there is a preauthorization process for targeted antibiotics at your CLC, which antibiotic agents are restricted in this manner? Please explain.

____

17d Please explain your "Other" response about policies and processes to improve antibiotic use.

____

18a Which of the following diagnostic stewardship processes for ordering urine cultures are in place at your CLC? Check all that apply.

- ☐ Urine cultures are reflexively ordered when certain urinalysis findings are abnormal
- ☐ Provider is prompted to choose a UTI symptom when placing the urine culture order
- ☐ Provider needs to specify how the urine culture was collected
- ☐ Other. Please explain below.
- ☐ None of the above.

18b Please explain your Other response about the diagnostic stewardship of urine cultures.

____

Education about antibiotic stewardship in your CLC

19a Does your facility provide education in your CLC about antibiotic resistance and opportunities for improving antibiotic use?

- ◯ Yes
- ◯ No

19b If your facility does provide educational resources about antibiotics to your CLC, indicate which of the following audiences have been engaged in the past year. Select all that apply.

- ☐ Clinical providers (e.g., MDs, NPs, PAs, PharmDs)
- ☐ Nursing staff (e.g., RNs, LPNs, CNAs)
- ☐ CLC patients and families
- ☐ Other. Please explain below.

19c Please explain your "Other" response about types of personnel you've educated.

____

Tracking and reporting in your CLC

20a Which of the following metrics does your CLC track to monitor antibiotic use and its downstream effects? Check all that apply.

- ☐ Adherence to clinical assessment documentation (signs/symptoms, vital signs, physical exam findings)
- ☐ Adherence to prescribing documentation (dose, duration, indication)
- ☐ Adherence to facility-specific treatment recommendations
- ☐ Point prevalence surveys of antibiotic use
- ☐ Rates of new antibiotic starts/1,000 resident-days
- ☐ Antibiotic days of therapy/1,000 resident-days
- ☐ C. difficile infections
- ☐ Infections with multidrug-resistant organisms
- ☐ Other. Please explain below.
- ☐ None of the above

20b Please explain your "Other" response about metrics you track.

____

21a Does your facility provide facility-specific reports on CLC-related antibiotic use and outcomes to CLC providers and/or CLC nursing staff?

- ◯ Yes
- ◯ No

21b Where is the CLC data shared? Select all that apply.

- ☐ Antibiotic stewardship committee meetings
- ☐ CLC staff meetings
- ☐ Leadership meetings
- ☐ Personalized feedback to providers
- ☐ Other. Please explain below.

21c Explain your "Other" response about where you share your facility-specific reports about antibiotic use and related outcomes.

____

Barriers to improving antibiotic use in your CLC

22a What, if any, barriers prevent improvements in antibiotic use at your CLC? Check all that apply.

- ☐ Lack of funding/resources for stewardship personnel to have dedicated time
- ☐ Hospital leadership has not made antibiotic stewardship a priority at our CLC
- ☐ CLC providers are resistant to input on their antibiotic-prescribing practices
- ☐ There are not personnel to develop a stewardship program in the CLC
- ☐ No access to an infectious disease expert
- ☐ Other. Please explain below.
- ☐ No barriers.

22b Please explain your "Other" response about barriers to improving antibiotic use in CLCs.

____
